# SkQ3 Exhibits the Most Pronounced Antioxidant Effect on Isolated Rat Liver Mitochondria and Yeast Cells

**DOI:** 10.3390/ijms25021107

**Published:** 2024-01-16

**Authors:** Anton G. Rogov, Tatyana N. Goleva, Dinara A. Aliverdieva, Renata A. Zvyagilskaya

**Affiliations:** 1 National Research Center “Kurchatov Institute”, 123182 Moscow, Russia; rogov_ag@nrcki.ru (A.G.R.); goleva_tn@nrcki.ru (T.N.G.); 2 Precaspian Institute of Biological Resources, Daghestan Federal Research Center of the Russian Academy of Sciences, 367000 Makhachkala, Russia; dinara_inbi@mail.ru; 3 A.N. Bach Institute of Biochemistry, Research Center of Biotechnology of the Russian Academy of Sciences, 119071 Moscow, Russia

**Keywords:** rat liver mitochondria, *Dipodascus magnusii* yeast, SkQ1, SkQ3, MitoQ, respiration, oxidative stress, cell death, mitochondrial fragmentation

## Abstract

Oxidative stress is involved in a wide range of age-related diseases. A critical role has been proposed for mitochondrial oxidative stress in initiating or promoting these pathologies and the potential for mitochondria-targeted antioxidants to fight them, making their search and testing a very urgent task. In this study, the mitochondria-targeted antioxidants SkQ1, SkQ3 and MitoQ were examined as they affected isolated rat liver mitochondria and yeast cells, comparing SkQ3 with clinically tested SkQ1 and MitoQ. At low concentrations, all three substances stimulated the oxidation of respiratory substrates in state 4 respiration (no ADP addition); at higher concentrations, they inhibited the ADP-triggered state 3 respiration and the uncoupled state, depolarized the inner mitochondrial membrane, contributed to the opening of the mPTP (mitochondrial permeability transition pore), did not specifically affect ATP synthase, and had a pronounced antioxidant effect. SkQ3 was the most active antioxidant, not possessing, unlike SkQ1 or MitoQ, prooxidant activity with increasing concentrations. In yeast cells, all three substances reduced prooxidant-induced intracellular oxidative stress and cell death and prevented and reversed mitochondrial fragmentation, with SkQ3 being the most efficient. These data allow us to consider SkQ3 as a promising potential therapeutic agent to mitigate pathologies associated with oxidative stress.

## 1. Introduction

Mitochondrial diseases, a pool of clinically heterogeneous pathologies [1,2], are caused by a wide range of factors, including mutations in the mitochondrial [3] or nuclear genome [1], leading to mitochondrial dysfunction in cells and tissues. Mitochondrial dysfunction is associated with impairment of the oxidative phosphorylation system [4,5]; however, some pathologies may not affect mitochondrial energy functions and are caused by defects in cell signaling systems [6,7], mitophagy processes [8,9] or programmed cell death [10].

A common factor in mitochondrial dysfunctions of different etiologies is oxidative stress [11,12,13], characterized by an increase in reactive oxygen species (ROS), generally as a result of an imbalance between their mitochondrial production and the activity of cell antioxidant systems. Mitochondrial reactive oxygen species (mROS) are a decisive factor in the mechanism underlying muscle atrophy and weakness (e.g., sarcopenia) [14] and may play a role in the pathogenesis of multiple sclerosis [15], age-related chronic diseases [16], type II diabetes associated with obesity [17] and senescence in mtDNA-mutant mice [18]. In long-lived neurons, where functionally active mitochondria must be maintained throughout life, the role of mROS has been hypothesized [19] as being critical to normal function and pathology (Alzheimer’s, Parkinson’s, motoneuron and Huntington’s diseases); in cancer cells, ROS production contributes to the development of more aggressive phenotypes [20]. Under severe oxidative stress, ATP levels are reduced; Ca^2+^ cytosol levels increase; cellular components, including DNA, proteins and lipids are damaged [21]; and multicellular signaling pathways are destroyed, resulting in the escalation of various pathologies [22,23] and induction of a program of cell death [24], which in multicellular organisms, is often associated with a systemic inflammation response [10,25]. Thus, reducing the generation of mROS and oxidative stress in cells could be a promising therapeutic strategy for the treatment of mitochondrial diseases [26,27,28,29].

The most promising in this respect could be mitochondrial-directed antioxidants, which are transported predominantly in the mitochondria (the sites of mROS formation). They should contain an antioxidant connected by a linker to a cation, which predetermines the transport of the compound in mitochondria according to the membrane potential, generated on the cytoplasmic and inner mitochondrial membrane (they are negatively charged) [30,31] As a result, their concentrations in mitochondria would increase by several orders of magnitude in comparison with the initial concentrations. If lipophilic mitochondrial-targeted antioxidants have a high distribution coefficient in the membrane, their concentration in the lipid bilayer may increase by several orders of magnitude. Moreover, if lipophilic mitochondria-targeted antioxidants have as an antioxidant ubiquinone (Q), the natural component of the electron transfer chain of mitochondria, they can be reduced (regenerated) by components of the respiratory chain, which ensures their repetitive functions. The first mitochondria-targeted antioxidant, MitoQ 10-(4,5-dimethoxy-2-methyl-3,6-dioxo-1,4-cyclohexadien-1-yl)decyltriphenylphosphonium) containing the positively charged lipophilic cation triphenylphosphonium bonded by a C 10 aliphatic chain with ubiquinone, was synthesized by the Murphy’s group [32].

Academician V. P. Skulachev replaced ubiquinone in the molecule of MitoQ with the potentially more powerful natural antioxidant plastoquinone, normally functioning in the photosynthetic electron transport chain (in chloroplasts) under severe conditions of elevated oxygen concentrations and ROS production [33]. Within the framework of a project supervised by Skulachev at the A.N. Belozersky Research Institute of Physico-Chemical Biology, MSU, Moscow, Russia, a series of plastoquinone derivatives (referred to collectively as SkQs, where Sk means “penetrating cation”, “Skulachev ion” is a term put forward by D. Green [34] and Q is plastoquinone) were synthesized. SkQ1 (10-(4,5-dimethyl-3,6-dioxo-1,4-cyclohexadien-1-yl)decyltriphenylphosphonium) was the first one [33].

SkQ1 and other cationic derivatives of plastoquinone were found to easily penetrate through bilayer lipid membranes forming a diffusion potential of the expected value [31]. They are selectively accumulated in cell mitochondria, prevent the oxidation of mitochondrial cardiolipin upon oxidative stress [35], protect from ROS-induced apoptosis or necrosis and protein carbonylation in aging animals, lengthen the lifespan of a wide circle of eukaryotes (from fungi to mammals) [36] and retard the development of approximately 30 typical symptoms of senescence and senile diseases [31,35,36,37,38,39,40,41].

Within the pool of mitochondrial-targeted antioxidants of the SkQ family, SkQ3 is a conjugate of methylplastoquinone and triphenylphosphonium, 10-(2,4,5-trimethyl-3,6-dioxo-1,4-cyclohexadien-1-yl)decyltriphenylphosphonium [31].

SkQ3 increases the lifespan of *Arabidopsis thaliana* rosette and reduces the oxidative stress during PAMP (pathogen-associated molecular pattern)-dependent immune responses in *Nicotiana tabacum* stomata cells [42]. Like other SkQ family compounds [33], SkQ3 increases the state 4 respiration supported by succinate in mitochondria isolated from the rat heart [31] and aerobic yeast *Dipodascus magnusii* [43]. This uncoupling activity of SkQ3 can be associated with the joint action of cationic antioxidant and endogenous fatty acids as it is almost totally abolished by BSA [31]. In isolated rat heart mitochondria, SkQ3, at very low concentrations, exhibits antioxidant activity, which is replaced by a weak prooxidant activity with an increasing concentration [31].

Although SkQ3 possesses antioxidant properties and has all the advantages of SkQs, to date, there has not been a comprehensive study on its effects on mitochondria. There is also no complete understanding of how SkQ properties can be related to the structure of their quinone fragment.

The study was mainly aimed to compare the properties of the less investigated compound SkQ with the better-studied SkQ and MitoQ, which are already therapeutic. Additionally, we wanted to understand whether there is a correlation between the structure of the quinone fragment of the substances used and their antioxidant properties. As models, we used isolated tightly-coupled rat liver mitochondria (RLM) and the yeast D. magnusii cells, marked by respiratory metabolism closely resembling that of mammalian cells [44,45] and vigorous growth on a variety of simple, well defined and inexpensive media; giant cells of this yeast make them a unique object for the visualization of mitochondria in a cell [46].

It was found that despite minimal differences in the structures, SkQ3 differs fundamentally from SkQ1 and MitoQ, lacking prooxidant activity at high concentrations, a property, which is of great importance for the potential therapeutic use of the substance. In this regard, SkQ3 is similar to other members of the SkQ family, SkQT1 and SkQThy, but without their shortcomings. In summary, the data obtained allow us to consider SkQ3 as a promising potential therapeutic agent to mitigate pathologies associated with oxidative stress.

## 2. Results

### 2.1. Effects of SkQ3, SkQ1 and MitoQ Supplementation on Isolated Rat Liver Mitochondria

In the first part of this study, we examined the mitochondria-targeted antioxidants SkQ3, SkQ1 and MitoQ and how they affect the functional parameters of the isolated tightly-coupled rat liver mitochondria (RLM), with a special focus on SkQ3, by comparing it with clinically tested SkQ1 and MitoQ [47,48,49].

**Figure 1:**
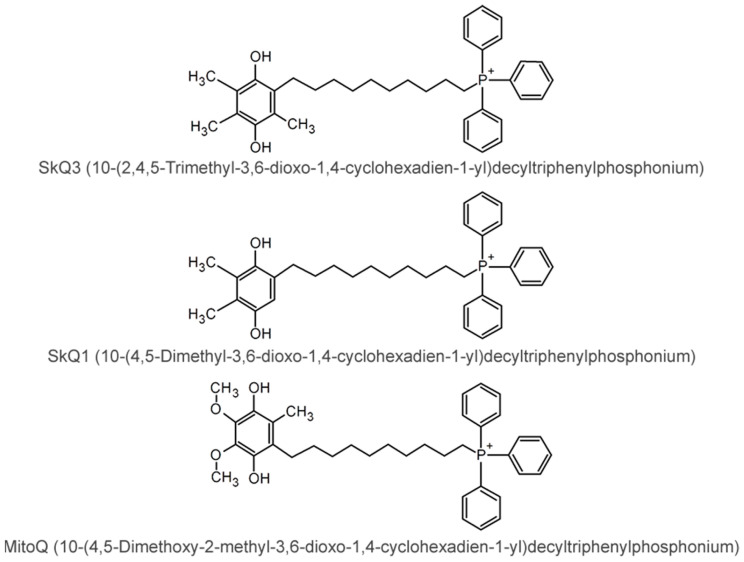
Structures of SkQ3, SkQ1 and MitoQ.

Figure 1 shows SkQ1, SkQ3 and MitoQ structures.

**Figure 2:**
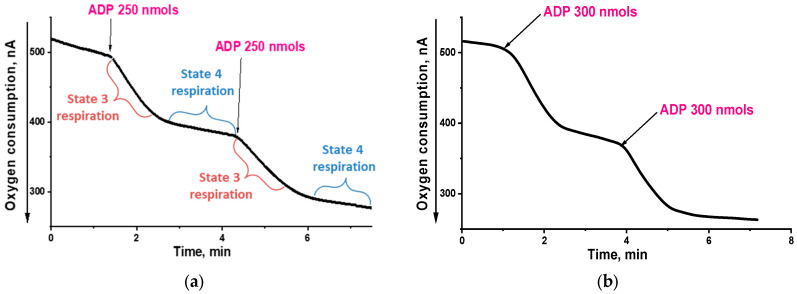
Amperometric recording of oxygen consumption by RLM oxidizing succinate (**a**) or glutamate and malate (**b**). The basic incubation medium containing 0.21 M mannitol, 0.09 M sucrose and 2 mM Tris-phosphate, pH 7.2, was supplemented with 0.5 mM EGTA, 20 mM Tris-succinate + 2 µg rotenone (**a**) or 20 mM Tris-glutamate + 5mM Tris-malate (**b**) and mitochondria corresponding to 0.5 mg protein/mL. Where indicated, ADP was added. Respiratory control values for successive ADP additives were (**a**) 8.3 and 8.9 and (**b***)* 7.3 and 9.8.

Isolated RLM used in this study met all the conventional and some additional criteria of physiological integrity, as evidenced by their ability to maintain distinctive state 4-to-3 respiration transitions upon successive additions of ADP (Figure 2a,b); high respiratory control values, calculated based on Chance and Williams [50,51], upon the oxidation of both succinate and NAD-dependent substrates (Figure 2a,b); ADP/O ratios upon the oxidation of succinate and NAD-dependent substrates approaching 2 and 3, respectively, which was close to the theoretically expected maxima for these substrates; the stimulation of state 4 respiration by AMP instead of ADP, indicating the presence of adenylate kinase in the mitochondrial intermembrane space and hence the intactness of the outer mitochondrial membrane; long retention of the maximal ΔΨ value in the absence of oligomycin, which proves the absence of proton leakage.

**Figure 3:**
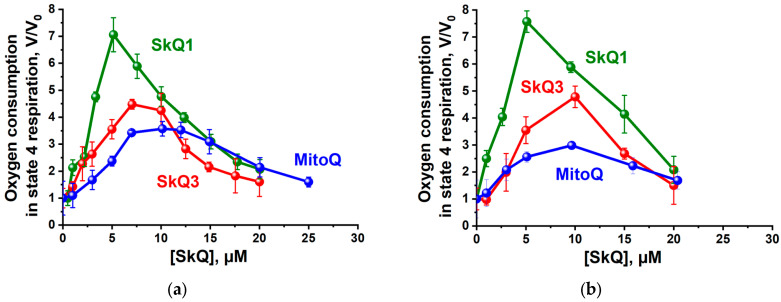
Effect of SkQ3, SkQ1 and MitoQ on oxygen consumption by rat liver mitochondria in the state 4 respiration oxidizing succinate (**a**) or glutamate and malate (**b**). The incubation medium was as in Figure 2. Data are presented as the mean ± S.D. from three replicates.

At low concentrations, all three substances studied stimulated the oxidation of succinate (Figure 3a) and NAD-dependent substrates (glutamate and malate) (Figure 3b) in state 4 respiration, which is a formal indicator of their uncoupling effect, most probably due to discharging of the membrane potential as a result of their electrophoretic movement into mitochondria and/or the cycling of endogenous fatty acids. SkQ1 was the strongest uncoupler, while SkQ3 exhibited (showed) a moderate uncoupling effect.

**Figure 4:**
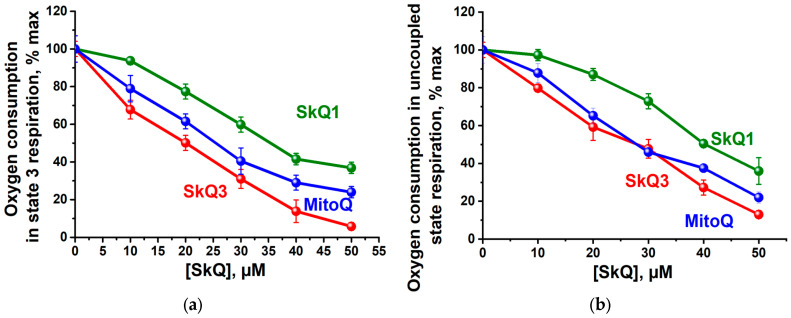
Effects of SkQ3, SkQ1 and MitoQ on oxygen consumption by RLM in the state 3 (**a**) or uncoupled state (**b**) of respiration supported by succinate. The basic incubation medium was supplemented with 0.5 mM EGTA, 20 mM Tris-succinate + 2 µg rotenone, mitochondria corresponding to 0.5 mg protein/mL and either 0.9 mM ADP (**a**) or 2 μM CCCP (**b**). State 3 respiration was initiated by adding 0.9 mM ADP, while the uncoupling state was triggered by the addition of 2 μM CCCP; 100% is defined as the respiratory rate before the addition of SkQ1, SkQ3 or MitoQ. Data are presented as the mean ± S.D. from three replicates.

At significantly higher concentrations, all three substances studied similarly inhibited state 3 respiration (in the presence of 0.9 mM ADP) (Figure 4a) and uncoupled respiration (in the presence of 2 μM carbonylcyanide *m*-chlorophenylhydrazone (CCCP) (Figure 4b) in mitochondria oxidizing succinate, thus suggesting the absence of the specific inhibition of ATP synthase or adenine nucleotide translocase. SkQ3 had a more pronounced inhibitory effect on state 3 or the uncoupled state of respiration, causing the same effect at lower concentrations.

**Figure 5:**
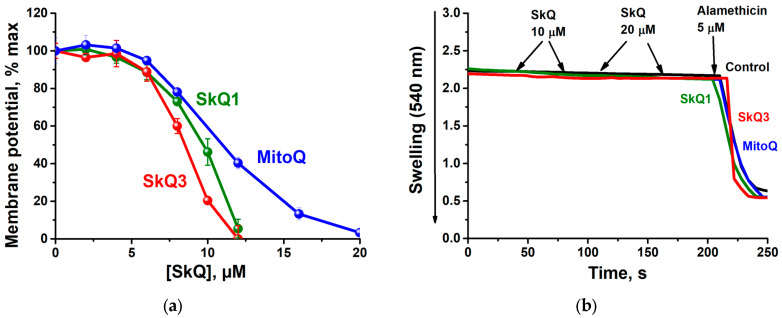
Effect of SkQ3, SkQ1 and MitoQ on the mitochondrial membrane potential (**a**) and swelling (**b**) of RLM oxidizing succinate. The basic incubation medium was supplemented with 0.5 mM EGTA, 20 mM Tris-succinate + 2 µg rotenone, mitochondria corresponding to 0.5 mg protein/mL and either 20 µM Safranine O (**a**) or 40 µM KCl and 5 µM Alamethicin (**b**). Where indicated, SkQ1, SkQ3 or MitoQ, respectively, were added. Data in (**a**) are presented as the mean ± S.D. from three replicates.

All three substances studied depolarized the inner mitochondrial membrane (Figure 5a). SkQ3 reduced the membrane potential more effectively than SkQ1 or MitoQ. None of the substances caused the high-amplitude swelling of mitochondria (Figure 5b), indicating that there was no detectable action of SkQ3, SkQ1 or MitoQ on nonspecific mitochondrial pores or ion channels. Alamethicin (5 μM), a hydrophobic 1 nm-channel-forming peptide, produced by the fungus *Trichoderma viride*, was used as a positive control.

**Figure 6:**
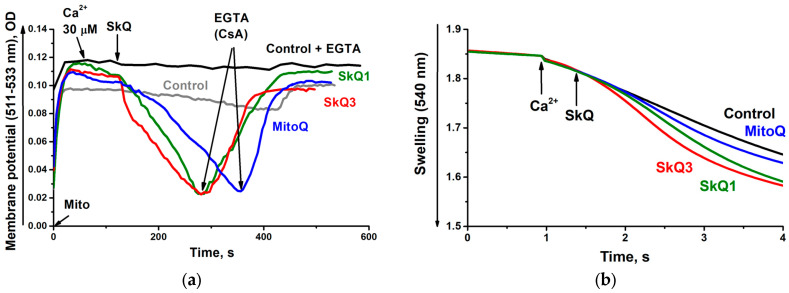
SkQ3, SkQ1 and MitoQ promote mPTP opening in RLM isolated and suspended in EGTA-free media. The basic Incubation medium was supplemented with 20 mM Tris-succinate + 2 µg rotenone, mitochondria corresponding to 0.5 mg protein/mL and either 20 µM Safranine O (**a**) or 40 µM KCl (**b**). Where indicated, 30 µM CaCl_2_, 0.5 mM EGTA or 1.8 µM CsA or 3 µM SkQ1, 3 µM SkQ3 or 3 µM MitoQ were added.

However, if the RLM were isolated and suspended in EGTA-free medium, the addition of 30 µM Ca^2+^ caused not only their spontaneous depolarization (Figure 6a) but also spontaneous swelling (Figure 6b). All three substances under study, taken in low (3 µM) concentrations, considerably increased the amplitude of these processes, most likely reflecting the promotion of the opening of the nonspecific Ca^2+^/phosphate-depended pore (the mPTP, permeability transition pore), as it was inhibited by EGTA or cyclosporine A (CsA) [52].

**Figure 7:**
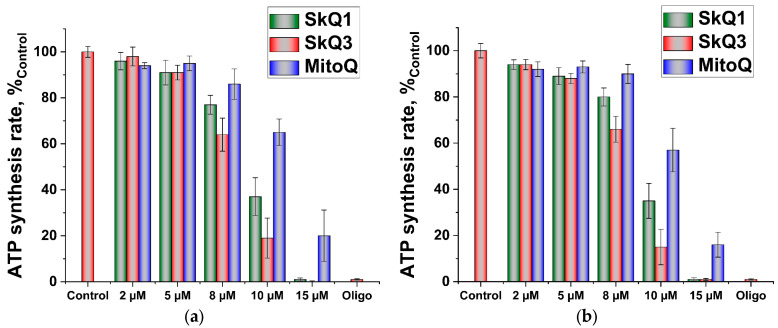
Effect of SkQ3, SkQ1 and MitoQ on ATP production by RLM oxidizing succinate. The basic incubation medium was supplemented with 0.5 mM EGTA, 20 mM Tris-succinate, rotenone (2 µg/mg), 1 µg/mL Ap5A, 20 µM Phenol Red (**a**) or 1 mM glucose + 2 mM NADP + 9U/mL hexokinase + 3U/mL glucose-6-phosphate dehydrogenase (**b**). Data are presented as the mean ± S.D. from three replicates.

SkQ3, SkQ1 and MitoQ lowered the rate of ATP synthesis by mitochondria, which was measured using two independent methods. The incubation medium was supplemented with 1 µg/mL P5-di(adenosine-5′)pentaphosphate (Ap5A), the inhibitor of adenylate kinase. The inhibition of ATP synthesis by all three tested substances, was in precise accordance with their depolarizing action (Figure 7), with SkQ3 exerting a more pronounced effect, thus supporting the earlier conclusion (see, Figure 4) about the minimal, if any, effects of these substances on ATP synthase and the translocase of adenine nucleotides.

**Figure 8:**
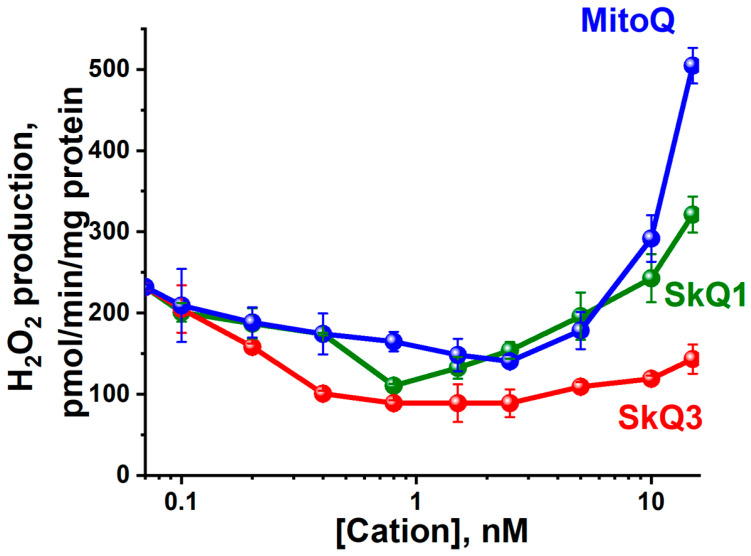
Effect of SkQ3, SkQ1 and MitoQ on peroxide production by RLM oxidizing succinate. The basic incubation medium was supplemented with 0.5 mM EGTA, 20 mM Tris-succinate, rotenone (2 µg/mg), 5 µM Amplex Red, 9 U horseradish peroxidase, 6 mM aminotriazole (an inhibitor of catalase) and mitochondria corresponding to 0.5 mg protein. Data are presented as the mean ± S.D. from three replicates.

Low, non-uncoupling concentrations of SkQ3, SkQ1 and MitoQ had a pronounced antioxidant effect, decreasing the hydrogen peroxide production by RLM (Figure 8). The basic incubation medium was supplemented with 6 mM aminotriazole (an inhibitor of catalase). Importantly, SkQ3 displayed the most considerable antioxidant effect and, unlike SkQ1 or MitoQ, did not exhibit prooxidant activity when the effective concentration was increased over the entire range studied. This SkQ3 property makes it one of the most promising mitochondria-targeted antioxidants with potential therapeutic action.

### 2.2. Effects of SkQ3, SkQ1 and MitoQ Suplementation on D. magnusii Cells

**Figure 9:**
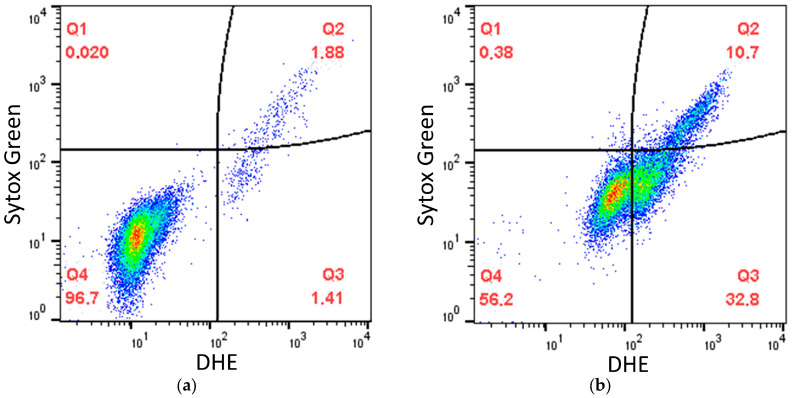
Oxidative stress and cell death in control (untreated) *D. magnusii* cells (**a**) and cells treated with *t*-BHP (**b**) for 2 h, as measured via flow cytometry. Q1—without cells; Q2—dead cells; Q3—cells with signs of oxidative stress; Q4—live cells with no signs of oxidative stress. The numbers inside the figure are the percentages of the prevailing subpopulation in the entire cellular population.

In the second part of this study, we tested the ability of SkQ3, SkQ1 and MitoQ to mitigate the oxidative stress induced by the classic prooxidant *tert*-butyl hydroperoxide (*t*-BHP) in *D*. *magnusii* cells. Yeast cells harvested in the early exponential growth phase were preincubated with different concentrations of SkQ3, SkQ1 and MitoQ and then transferred to the medium with *t*-BHP and stained with two dyes: dihydroethidium (DHE), detecting intracellular reactive oxygen species (ROS) (with increased affinity for the superoxide anion radical), and Sytox Green, staining only dead cells with a damaged cytoplasmic membrane. The fluorescence of double-stained yeast cells was examined via flow cytometry. Figure 9 shows (as an example) that the entire cell population was divided into three subpopulations according to the intensity of dye fluorescence. The subpopulation with low fluorescence of both DHE and Sytox Green (Q4) corresponded to living cells with no signs of oxidative stress, not subjected to oxidative stress (for short, “normal” cells); cells with increased DHE fluorescence (Q3) were thought to be alive with oxidative stress; the subpopulation of cells with high fluorescence of both dyes (Q2) represented dead cells (Figure 9 as an example). A two-hour incubation of yeast with *t*-BHP increased the level of intracellular oxidative stress and cell death.

**Figure 10:**
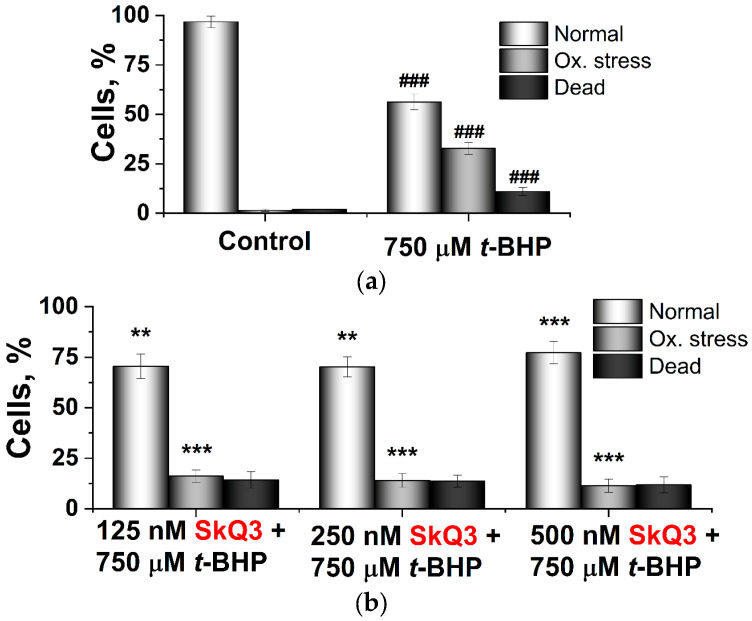

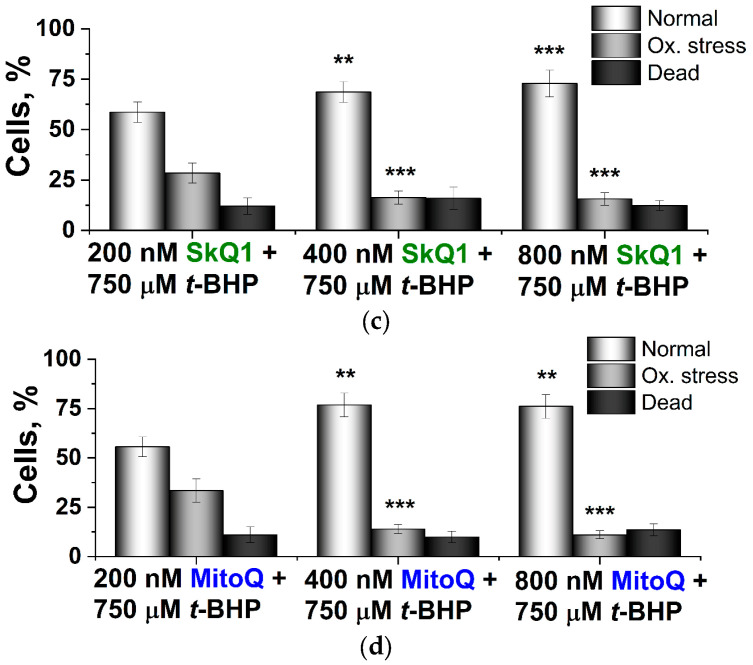
The oxidative stress and cell death in *D. magnusii* yeasts treated with *t*-BHP for 2 h (**a**). Effects of SkQ3 (**b**), SkQ1 (**c**) and MitoQ (**d**). Results were obtained via flow cytometry measurements from three independent experiments, presented as histograms. Statistical analyses were carried out via the one-way ANOVA test. Symbols: * marks differences between the results obtained for SkQ-preincubated samples against only *t*-BHP-treated ones; ***: *p* < 0.001, **: 0.001 < *p* < 0.01; # marks differences between the control and *t*-BHP-treated sample; ###: *p* < 0.001.

Figure 10 shows the processed data as histograms. The preincubation of yeast cells with all three mitochondria-targeted antioxidants reduced intracellular oxidative stress and cell death, albeit with different efficacies (Figure 10b–d), with the most effective SkQ3 causing the most pronounced protective action, at lower concentrations.

**Figure 11:**
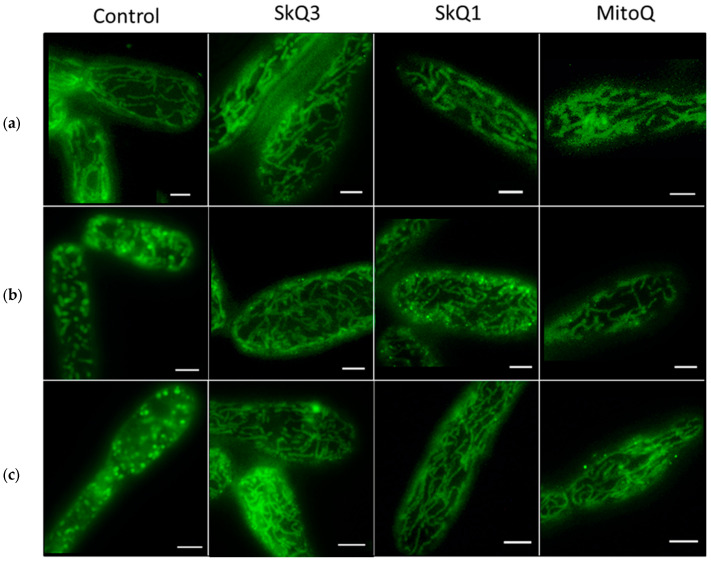
*t*-BHP-induced fragmentation of mitochondria in *D. magnusii* cells and protective effects of SkQ3, SkQ1 and MitoQ on the integrity of the mitochondrial reticulum. (**a**) No preincubation with *t*-BHP; (**b**) where indicated, cells were preincubated with 250 nM SkQ3, 800 nM SkQ1 or 800 nM MitoQ for 1 h and then incubated with 750 μM *t*-BHP for 2 h; (**c**) where indicated, cells were preincubated with 250 μM *t*-BHP for 2 h and then incubated with 250 nM SkQ3, 800 nM SkQ1 or 800 nM MitoQ for 1 h. Bars are 5 μm.

Normally, *D. magnusii* cells possess a branched mitochondrial reticulum (Figure 11a, Control), which, however, is fragmented upon the induction of mitochondrial dysfunction (inhibition of respiration and uncoupling) or oxidative stress [46]. The incubation of “normal” *D. magnusii* cells with SkQ3, SkQ1 and MitoQ did not alter the morphology of mitochondria (Figure 11a, Control). The treatment of cells with low *t*-BHP concentrations led to complete mitochondrial reticulum fragmentation (Figure 11b, Control). Moreover, fragmented mitochondria did not regenerate into the mitochondrial network even after removing the prooxidant from the incubation medium (Figure 11c, Control). Importantly, cell preincubation with low concentrations of all three tested antioxidants prevented mitochondrial fragmentation when yeast cells were subsequently exposed to the prooxidant (Figure 11b, SkQ3, SkQ1, MitoQ). It is even more astonishing that the addition of low concentrations of SkQ3, SkQ1 and MitoQ to cells with fragmented mitochondria led to the recovery of the mitochondrial reticulum. Based on the data obtained, SkQ3, SkQ1 and MitoQ were effective in preventing and returning the mitochondrial fragmentation caused by oxidative stress. Once again, SkQ3 was the most effective in these two processes, as its preventing or recovering concentrations were the lowest.

## 3. Discussion

A thorough analysis of the effects of SkQ1, SkQ3 and MitoQ on RLM revealed only minor differences in the decoupling effects (Figure 3), respiratory inhibition (Figure 4), membrane depolarization (Figure 5a), the promotion of mPTP (mitochondrial permeability transition pore) (Figure 6) or impact on the rate of ATP synthesis (Figure 7). More surprisingly, the most pronounced antioxidant effect of SkQ3 on RLM seemed to be shown in the entire range of concentrations studied (Figure 8). Previously, such effects were observed only for SkQT1 ((toluquinonyl)decyltriphenylphosphonium) and SkQThy ((thymoquinonyl)decyltriphenylphosphonium), derivatives of toluquinone and thymoquinone, respectively [27,29], and the absence of a prooxidant effect at high concentrations was considered to be a great advantage of the substance as a potential therapeutic agent [27,29]. However, the main drawbacks of SkQT1 were the relatively low stability and the presence of a mixture of two isoforms in the ratio of 1.4 : 1 [29]. SkQThy is also a mixture of two isoforms [27], which makes it difficult to assess the contribution of each of the isoforms to overall antioxidant activity and their possible application in therapy.

In the tested yeast model, *D. magnusii*, we were able to not only confirm the data, obtained on mitochondria, but also quantify and compare the antioxidant effects of all three mitochondria-targeted antioxidants used (Figure 10, flow cytometry), as well their ability to prevent mitochondrial fragmentation under induced oxidative stress and, moreover, to restore the mitochondrial reticulum (Figure 11). The optimal concentrations of SkQ3 in these processes were much lower than those of SkQ1 and MitoQ. The cumulative data obtained suggest that SkQ3 is one of the most effective mitochondria-targeted antioxidants and does not have the disadvantages of SkQT1 and SkQThy.

As for the correlation between the quinone fragment structure and antioxidant activity of the examined mitochondria-targeted antioxidants, this is not so clear. MitoQ, a ubiquinone conjugate, had lower antioxidant activity compared to SkQ1 [30], while SkQ1, a plastoquinone conjugate, had lower antioxidant activity compared to the other plastoquinone conjugates SkQT1 and SkQThy [27,29], as well as SkQ3 (Figure 8, this study).

MitoQ, with relatively weak antioxidant activity, and SkQ3 (high activity, this study) both have no substituted atoms in the quinone fragment of the molecule, while SkQThy (high activity) and SkQ1 (moderate activity) have one and SkQT1 (high activity) has two unsubstituted atoms in the quinone ring.

Thus, we can conclude that there is no clear (obvious) correlation between the structure of the quinone ring (more precisely, the number of substituted atoms) in the molecules of the mitochondria-targeted antioxidants and their antioxidant activities, contrary to what has been asserted previously [33].

## 4. Materials and Methods

### 4.1. Reagents

ADP, Alamethicin, 3-amino-1,2,4-triazole, P1,P5-di(adenosine-5′)pentaphosphate (Ap5A), Amplex Red, ATP, carbonyl cyanide *m*-chlorophenylhydrazone (CCCP), cyclosporine A (CsA), EGTA, fatty acid-free BSA, glucose, glucose-6-phosphate dehydrogenase, glutamate, hexokinase, horseradish peroxidase, malate, mannitol, MgCl_2_, NaCl, NADP, (NH_4_)_2_SO_4_, oligomycin, Phenol Red, phosphoenolpyruvate, pyruvate kinase, rotenone, succinate, sucrose, *tert*-butyl hydroperoxide and Tris were from Sigma-Aldrich (Saint Louis, MO, USA); Coomassie G-250 was from MP Biomedicals (Santa Ana, CA, USA); CaCl_2_, KCl, K_2_HPO_4_, KH_2_PO_4_ and Safranine O were from Merck (Darmstadt, Germany); Dihydroethidium, Mitotracker Green FM, Propidium Iodide and Sytox Green were from Life Technologies (Carlsbad, CA, USA). Other reagents of the highest purity available were from domestic suppliers. MitoQ, SkQ1 and SkQ3 were kindly provided by Dr. D.S. Esipov from the A.N. Belozersky Research Institute of Physico-Chemical Biology, MSU, Moscow, Russia.

### 4.2. Isolation of RLM

All manipulations with animals were performed in accordance with the Guide for the Care and Use of Laboratory Animals [53] approved by the Local Ethics Committee of the National Research Center “Kurchatov Institute” (protocol no. 1 of 20 December 2022). Two-to-three-month-old (200–250 g) outbred Wistar male rats were used in this study. The animals were fed ad libitum and had full access to tap water. They were killed via decapitation. RLM were isolated via differential centrifugation as described previously [27] with minor modifications. Unless otherwise specified, the homogenization medium contained 0.21 M mannitol, 0.09 M sucrose, 10 mM Tris–HCl buffer, pH 7.2, and 0.5 mM EGTA; the homogenate was centrifuged at 6600× g for 12 min; the mitochondrial pellet was washed with the same buffer and resuspended in a minimum volume of the same buffer. All isolation procedures were conducted at 4 °C. The final mitochondrial preparations were kept on ice and remained fully active for at least 4 h.

### 4.3. Mitochondrial Protein Assay

The mitochondrial protein was assayed using the Bradford method [19] with BSA as a standard.

### 4.4. Oxygen Consumption Assay

Oxygen consumption by RLM was monitored amperometrically using a Clark-type oxygen electrode as described in [27]. The basic incubation medium containing 0.21 M mannitol, 0.09 M sucrose and 2 mM Tris–HCl buffer, pH 7.2, was supplemented with 20 mM Tris-succinate + rotenone (2 µg/mg protein) or 20 mM Tris-glutamate + 5 mM Tris-malate, 0.5 mM EGTA and mitochondria (0.5 mg protein/mL). Respiratory control values were calculated according to [54].

### 4.5. Transmembrane Potential Measurement

The membrane potential (ΔΨ) generated on the inner mitochondrial membrane of RLM was recorded spectrophotometrically using a DU-650 spectrophotometer (Beckman Coulter, Brea, CA, USA) [55]. The basic incubation medium was supplemented with 20 mM Tris-succinate + rotenone (2 µg/mg protein) or 20 mM Tris-glutamate + 5 mM Tris-malate, 0.5 mM EGTA, 20 µM Safranine O (as a ΔΨ-related probe) and mitochondria (0.5 mg protein/mL).

### 4.6. Swelling of RLM

Mitochondrial swelling was monitored spectrophotometrically using a Cary 300 Bio spectrophotometer (Varian, Palo Alto, CA, USA) as a decrease in the apparent absorbance of the mitochondrial suspension at 540 nm [27]. The basic incubation medium was supplemented with 20 mM Tris-succinate + rotenone (2 µg/mg protein), 40 mM KCl, mitochondria (0.5 mg protein/mL) and, where indicated, with 0.5 mM EGTA and/or 1.8 µM CsA.

### 4.7. Opening of the Nonspecific Ca^2+^/P_i_-Dependent Pore

Opening of the nonspecific Ca^2+^/Pi-dependent pore (mPTP) was detected based on a combination of both the membrane potential depolarization and large-scale swelling of mitochondria isolated and incubated in EGTA-free medium, as recommended in [52].

### 4.8. ATP Synthesis by RLM

The ATP synthesis rate was monitored spectrophotometrically using two methods: based on a pH shift during ADP conversion to ATP using Phenol Red as an indicator with a DU-650 spectrophotometer (Beckman Coulter, Brea, CA, USA) at 557/618 nm or based on NADP reduction at 340 nm in the coupled reactions catalyzed by hexokinase and glucose-6-phosphate dehydrogenase with a Cary 300 Bio spectrophotometer (Varian, Palo Alto, CA, USA) [27]. The basic incubation medium was supplemented with 0.5 mM EGTA, 6 µM Ap5A (the inhibitor of adenylate kinase) and mitochondria (0.5 mg mitochondrial protein/mL). In the first case, additionally added 25 µM Phenol Red (pH-dependent dye) and all stock solutions and additives were carefully adjusted to pH 7.1; the synthesis of ATP was induced by the addition of 500 µM ADP. In the second case, 1 mM glucose, 1 mM NADP, hexokinase (10 IU/mL) and glucose-6-phosphate dehydrogenase (3 IU/mL) were added, and the synthesis of ATP was initiated with 100 µM ADP.

### 4.9. Hydrogen Peroxide Production by RLM

The rate of hydrogen peroxide generation by RLM was determined fluorometrically with a RF 5301PC fluorescence spectrophotometer (Shimadzu, Kyoto, Japan) based on the Amplex Red/horseradish peroxidase (HRP) assay. The superoxide-anion radical initially formed by mitochondria is converted spontaneously or via the action of superoxide dismutases to hydrogen peroxide, which oxidizes the colorless substance Amplex Red into highly fluorescent resorufin (excitation and emission wavelengths (ex and em) were set to 563 and 587 nm, respectively). The assay was calibrated with known H_2_O_2_ concentrations. The basic incubation medium was supplemented with 20 mM Tris-succinate, 5 µM Amplex Red, horseradish peroxidase (9 IU/mL) and 6 mM aminotriazole (an inhibitor of catalase).

### 4.10. Yeast Cell Culture

Cells of the yeast *Dipodascus magnusii*, strain VKM Y261, were cultivated in liquid glycerol-containing medium at 28 °C with agitation (220 rpm) [56] and harvested at the early exponential growth phase (OD_590_ = 1.0).

### 4.11. Visualization of Mitochondria in Cells

For mitochondria visualization, yeast cells were stained with the fluorescent probe MitoTracker Green FM, which labels mitochondria in a membrane potential-independent manner [57]. Stained cells were observed under a Axioskop 40 fluorescence microscope (Zeiss, Oberkochen, Germany).

### 4.12. ROS Generation and Determination

Oxidative stress in yeast cells was induced by *tert*-butyl hydroperoxide (*t*-BHP), a well-known model prooxidant, and detected with 10 μM dihydroethidium (DHE) as a ROS marker. Cells were incubated with 10 μM DHE for 30 min at room temperature in the dark. The fluorescence of DHE was measured via flow cytometry as described in [58], via flow cytometry with a FACSCalibur flow cytometer (Becton Dickinson, Franklin Lakes, NJ, USA).

### 4.13. Cell Viability Assay

Cell viability was assayed with 10 μM Sytox Green. The fluorescence of Sytox Green was measured via flow cytometry as described in [59].

### 4.14. Statistical Analysis

All experiments with yeast mitochondria were performed at least three times. For the analysis of the mitochondrial morphology, at least fifty cells were examined in each trial. Statistical analyses were performed using one-way ANOVA with the post hoc Tukey HSD test. Data are presented as the mean ± S.D. from at least three independent replicates.

## Data Availability

The data used to support the findings of this study are available from the corresponding author upon request.
